# Expectation of life at old age: revisiting Horiuchi-Coale and reconciling with Mitra

**DOI:** 10.1186/s41118-018-0029-7

**Published:** 2018-02-04

**Authors:** Dalkhat M. Ediev

**Affiliations:** 1 International Institute for Applied Systems Analysis, Wittgenstein Centre for Demography and Global Human Capital (IIASA, VID/ÖAW, WU), Schlossplatz 1, 2361 Laxenburg, Austria; 2 Institute for Applied Mathematics and Information Technologies, North-Caucasian State Humanitarian-Technological Academy, Stavropolskaya 36, Cherkessk, 369000 Russia; 3 Demography Chair (HSMSS), Lomonosov Moscow State University, Leninskie Gory 51, r. 752, Moscow, 119992 Russia

**Keywords:** Old-age mortality, Life expectancy, Life table, Motality estimation, Age exaggeration

## Abstract

Data quality issues at advanced old age, such as incompleteness of registration of vital events and age misreporting, compromise estimates of the death rates and remaining life expectancy at those ages. Following up on Horiuchi and Coale (Population Studies 36: 317-326, 1982), Mitra (Population Studies 38: 313-319, 1984, Population Studies 39: 511–512, 1985), and Coale (Population Studies 39: 507–509, 1985), we examine the conventional approaches to constructing life tables from data deficient at advanced ages and the two adjustment methods by the mentioned authors. Contrary to earlier reports by Horiuchi, Coale, and Mitra, we show that the two methods are consistent and useful in drastically reducing the estimation errors in life expectancy as compared to the conventional approaches, i.e., the classical open age interval model and extrapolation of the death rates. Our results suggest complementing the classical estimates of life expectancy by adjustments using Horiuchi-Coale, Mitra, or other appropriate methods and avoiding the extrapolation method as a tool for estimating the life expectancy.

## Introduction

The life table model, which describes the current mortality profile in terms of a hypothetical survival of individuals in a synthetic cohort, is an important tool in studying mortality and its many implications such as insurance policies, social policies, and population projections (Chiang 1978; Preston et al. 2001). It builds upon age-specific death rates and produces various indicators of mortality, survival, and longevity for the age groups. A limitation of the model appears at the older ages where data scarcity or deficiency forces statisticians to disregard age details and aggregate the available data into a single “open age interval” (Missov et al. 2016).

Typical data problems that prevent the extension of the life table to an older age are age exaggeration and other types of age misreporting, such as poorly documented return migration following retirement and missing death records. A recent study by Randall and Coast (2016) suggests that the data quality at ages 60+ in low-income countries is yet “very rough” with only a limited improvement over time in African countries. Even in developed countries with generally good mortality data, incompleteness of migrant registration or misreported ages at death may bias the mortality estimates for the elderly (Khlat and Courbage 1996; Kibele et al. 2008; Preston et al. 1996). In regional demography and demography of social groups, small population size may be another source of data limitation that demands lowering the age at the start of the open age interval (Scherbov & Ediev 2011).

Choosing a broader open age interval that begins at younger ages may help mitigate some of the data quality problems, including the problems of age exaggeration and small population size. Lowering the age at the beginning of the open age interval, however, may itself have severe consequences for the quality of life table estimates because of departures from stationarity which is conventionally assumed for the age composition at the open age interval (Preston et al. 2001). In a stationary population, life expectancy at the beginning of the open age interval is inverse to the death rate:

1 $$ {e}_a={M_{a+}}^{-1} $$

Hereinafter, *a* denotes the starting age of the open age interval, *e*_*a*_ is the life expectancy at age *a*, and *M*_*a*+_ is the death rate in the open age interval. In this paper, we call (1) *the classical estimate (approach)*.

The classical approach is not the only one available for cases with age reporting problems. The most common alternative method to calculate the last “problematic” age range of the life table is *extrapolation* of death rates based on their change at younger ages in combination with a mortality model (Mathers and Ho 2014; Missov et al. 2016; UN DESA/Population Division 1982, 2017; Wilmoth et al. 2007). The World Health Organization (WHO) (Mathers and Ho 2014) used to extrapolate the death rates above the age of 85 by assuming a logistic mortality model. The United Nations (UN DESA/Population Division 1982) construct life tables for developing countries by fitting the Gompertz-Makeham model at younger ages and closing at age 85+. The Human Mortality Database (HMD) (Wilmoth et al. 2007) also corrects the original data at ages 80+. Extrapolation does not assume population stationarity or any other population model. Such extrapolation, however, ignores the original empirical data pertaining to the open age interval. Furthermore, as we demonstrate in this paper, the extrapolation method tends to be less accurate in terms of life expectancy than the other methods considered here.

Two other alternatives to the classical approach also work with the open age interval, just like the classical method, but relax the stationarity assumption presumed in (1). Indeed, populations are rarely stationary. Improving survival and changing fertility and immigration modify the population age composition. Growing populations are typically of younger age composition, with more weight on younger ages with lower mortality (Preston et al. 2001); their death rate in the open age interval is lower than in a stationary population with similar mortality. As a result, the classical method is prone to overestimating life expectancies for such populations. To account for effects of population growth, Horiuchi and Coale (1982) assumed the stable population model (Preston et al. 2001) with the Gompertzian mortality (Gompertz 1825; Heligman and Pollard 1980) to develop the following formula:

2 $$ {e}_a={M_{a+}}^{-1}{e}^{-{\beta}_ar{M_{a+}}^{-{\alpha}_a}} $$

Here, *r* is the annual growth rate of the population in the open age interval and *α*_*a*_ and *β*_*a*_ are the model parameters (for numerical values, see Horiuchi and Coale 1982 or Ediev 2017).

Adjustment (2) was challenged by Mitra (1984, 1985) who also assumed population stability and derived a closed form solution:

3 $$ {e}_a={M_{a+}}^{-1}{e}^{-r\left[{M_{a+}}^{-1}-\left(1+r{M_{a+}}^{-1}\right)\left(\overline{x}-a\right)\right]}, $$

where $\overline{x}$ stands for the mean age of the population in the open age interval. Mitra’s approach was, however, criticized for being prone to biases due to age exaggeration (Coale 1985). Indeed, having age exaggeration as a problem in the first place, one would cautiously use the empirical mean population age $\overline{x}$ in an adjustment procedure.

Partly because the discussion between Horiuchi-Coale and Mitra was never resolved, but also due to the strong assumptions used in both approaches, their methods have not made it to a wider practical use by demographers and population statisticians.

Two developments since the time of discussion between Horiuchi-Coale and Mitra call for revisiting their results. First, the empirical basis for mortality studies and computational resources has advanced substantially, as it is reflected in the rich collection of high-quality data in the HMD (2017). Second, life expectancy in many countries has systematically advanced since 1980. On the one hand, better survival to old age boosts the importance of the open age interval for life table estimates. On the other hand, higher (and improving) life expectancies call for testing if the old methodology works well on current data. Our paper is a response to these needs and introduces some useful modifications to the original adjustment formulas. We also aim at reconciling the dispute between Horiuchi-Coale and Mitra and combining alternative methods for a better outcome.

## Testing the models of expectation of life at old age on empirical data

To examine the biases in estimated life expectancy for selected estimation methods at different open age intervals, we use all period life tables, single-year mortality data, and population exposures contained in the HMD (2017).^1^ Altogether, the database contains 4436 country-calendar years for each gender (males, females, total), for 46 countries/populations, spanning over the years 1751 to 2014 and life expectancies at birth from 16.7 to 86.4 years. For each of the 3 × 4436 database entries, we recalculate its life table assuming alternative open age intervals (the threshold age *a* spanning from 55 to 95 years) and various estimation methods.

For each open age interval, we calculate the aggregated death rate for the open age interval using the death rates and population exposures from the HMD:

4 $$ {M}_{a+}=\frac{\sum_{x=a}^{\omega }{M}_x{P}_x}{\sum_{x=a}^{\omega }{P}_x} $$

Here, *M*_*x*_ and *P*_*x*_ are the HMD death rate and the population exposure at age *x*, respectively, and *ω* is the maximum attainable age group (110+ in HMD). After obtaining the death rate for the open age interval, we apply alternative life table methods and compare the results to the life table that is based on the full age scale spanning up to the age 110+.

First, we apply the classical life table method (1) and use estimates (4) and the death rates at ages below age *a* to calculate a new “truncated” life table for each HMD entry. Life expectancy from the truncated life table is compared to life expectancy from the full life table, where the “full” life table stands for the life table with the maximum possible *a* = 110. The difference between the two gives the estimation error in the classical life table method due to the truncation of the data at the selected open age interval.

In a similar fashion, we examine the estimation errors in the methods of Horiuchi-Coale (2) and Mitra (3) and in the extrapolation method where death rates are extrapolated into the open age interval based on their rate of increase at younger age. We improved the stability of the Horiuchi-Coale and Mitra formulas by using the population growth rates averaged over 10-year periods prior to the estimation year. If we used the annual rates (results not presented here), the adjusted life expectancies would contain more outliers, especially in the Mitra method. For extrapolations, we use the Gompertz model (Doray 2008; Gompertz 1825) where the force of mortality increases exponentially with age. After some experimentation, we opt for the marginally better extrapolation without jump of the death rate at age *a*, with parameters fit on 20-years-of-age-long age intervals below the open age interval. This extrapolation is close to the common practices for low-quality data cases. The Gompertz model is also useful as a bridge to the work by Horiuchi-Coale who relied on the model in developing their own method.

**Fig. 1:**
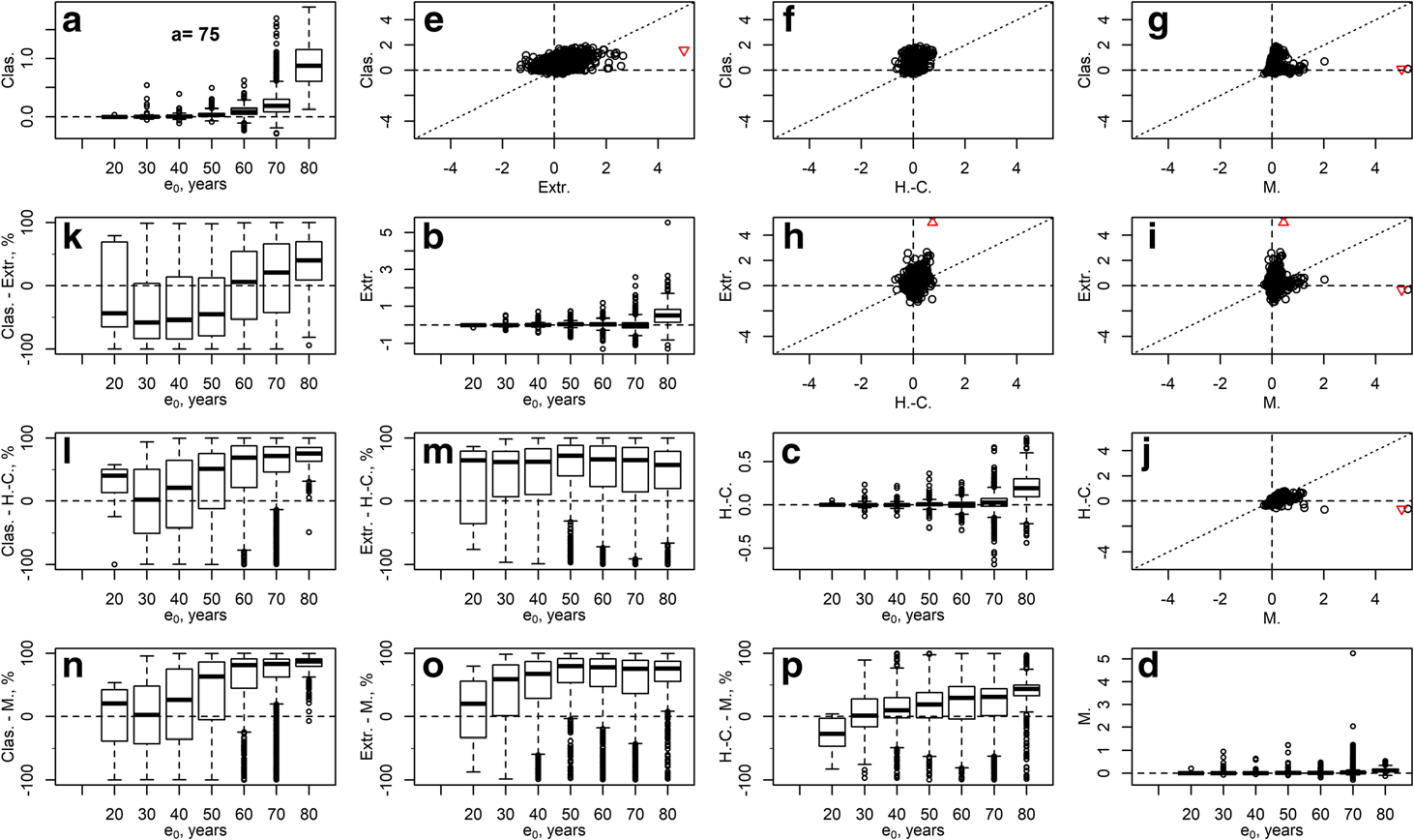
Estimation errors in life expectancy at birth obtained by methods: the classical method (“Clas.”), extrapolation based on 20-years-long age base (“Extr.”), Horiuchi-Coale method (“H.-C.”), and Mitra method (“M.”). The main diagonal (plots **a**–**d**): boxplots, as a function of life expectancy at birth, of estimation errors in each of the methods; above the diagonal (plots **e**–**j**): scatterplots of errors in pairs of methods as indicated at axes of the plots; below the diagonal (plots **k**–**p**): boxplots, as a function of life expectancy at birth, of percentage differences of absolute errors (5) of pairs of methods as indicated at vertical axes. Female populations, open age interval set at 75+

**Fig. 2:**
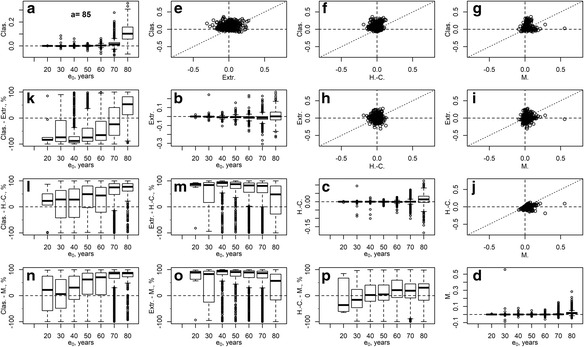
Estimation errors in life expectancy at birth obtained by methods: the classical method (“Clas.”), extrapolation based on 20-years-long age base (“Extr.”), Horiuchi-Coale method (“H.-C.”), and Mitra method (“M.”). The main diagonal (panels **a**–**d**): boxplots, as a function of life expectancy at birth, of estimation errors in each of the methods; above the diagonal (plots **e**–**j**): scatterplots of errors in pairs of methods as indicated at axes of the plots; below the diagonal (plots **k**–**p**): boxplots, as a function of life expectancy at birth, of percentage differences of absolute errors (5) of pairs of methods as indicated at vertical axes. Female populations, open age interval 85+

Comparative results for estimation errors of life expectancy at birth and age *a* in all methods are presented in Figs. 1 and 2 and Table 1. The table contains root-mean squared error (RMSE) of the life expectancy at birth and percentage RMSEs of the life expectancy at age *a* at open age intervals 55+, 65+, 75+, 85+, and 95+, for female, male, and total populations. Figures 1 and 2 present comparative results for estimation errors of the life expectancy at birth in female populations and open age intervals, 75+ and 85+, respectively. Plots in the main diagonal of each figure present boxplots of estimation errors of each of the methods as a function of the life expectancy at birth in the full life table. Scatterplots above the main diagonal allow comparing the errors in pairs of the methods. Boxplots below the main diagonal present distributions of the percentage differences in absolute errors between the pairs of the methods as functions of life expectancy at birth from the full life table, in percent of the higher absolute error:

5 $$ \mathrm{Perc}.\mathrm{Diff}.=\frac{\mathrm{abs}\left({\mathrm{err}}_1\right)-\mathrm{abs}\left({\mathrm{err}}_2\right)}{\operatorname{MAX}\left(\mathrm{abs}\left({\mathrm{err}}_1\right),\kern0.5em \mathrm{abs}\left({\mathrm{err}}_2\right)\right)}. $$

**Table 1:** Root-mean squared errors (RMSEs) in life expectancy at birth *e*_0_, percentage RMSEs in life expectancy in the open age interval *e*_*a*_ (by method): by sex, level of life expectancy at birth, and open age interval (*a*+)

			RMSE in *e*_0_ by method (years)						Percentage RMSE in *e*_*a*_ by method (percent)					
Sex	*e*_0_ range	*a*	Classical	Extrapol.	H.-C.	H.-C. (hmd)	Mitra	Mitra (regr.)	Classical	Extrapol.	H.-C.	H.-C. (hmd)	Mitra	Mitra (regr.)
Female	40–50	55	0.75	6.60	0.40	0.41	0.47	0.46	8.05	73.76	4.54	4.64	5.33	5.22
Female	40–50	65	0.25	0.70	0.17	0.17	0.23	0.22	5.17	15.61	3.67	3.71	4.83	4.72
Female	40–50	75	0.06	0.16	0.05	0.05	0.05	0.05	3.40	8.42	2.78	2.85	2.93	2.88
Female	40–50	85	0.01	0.03	0.01	0.01	0.02	0.02	2.21	9.50	2.29	2.36	3.03	2.65
Female	50–60	55	1.53	10.23	0.57	0.54	0.38	0.48	11.86	81.82	4.54	4.31	3.14	3.90
Female	50–60	65	0.53	1.45	0.23	0.23	0.21	0.24	7.65	21.53	3.41	3.38	3.07	3.51
Female	50–60	75	0.12	0.25	0.07	0.08	0.08	0.08	4.54	12.93	2.82	2.85	2.73	2.89
Female	50–60	85	0.01	0.04	0.01	0.01	0.01	0.01	2.60	7.94	2.00	2.04	2.08	2.09
Female	60–70	55	3.57	18.78	0.79	0.77	0.43	0.57	21.21	114.15	4.63	4.59	2.52	3.34
Female	60–70	65	1.23	1.56	0.29	0.29	0.19	0.25	12.99	17.34	3.11	3.12	1.99	2.65
Female	60–70	75	0.25	0.29	0.09	0.09	0.06	0.09	6.55	9.15	2.54	2.53	1.77	2.30
Female	60–70	85	0.02	0.06	0.01	0.01	0.01	0.01	3.11	8.14	1.95	1.95	1.81	1.97
Female	70–80	55	5.38	3.32	1.36	1.19	0.99	1.08	24.14	15.00	5.99	5.29	4.53	4.93
Female	70–80	65	2.33	2.18	0.49	0.48	0.37	0.42	16.87	15.72	3.53	3.48	2.70	3.06
Female	70–80	75	0.67	0.56	0.16	0.15	0.11	0.13	10.10	8.25	2.56	2.35	1.75	2.14
Female	70–80	85	0.09	0.10	0.03	0.03	0.02	0.03	4.78	7.20	1.70	1.72	1.34	1.57
Female	80–90	55	8.08	23.67	1.90	1.61	0.96	1.17	28.64	81.77	6.86	5.81	3.50	4.22
Female	80–90	65	3.78	4.71	0.91	0.89	0.64	0.74	20.07	25.14	4.91	4.79	3.45	3.95
Female	80–90	75	1.42	2.11	0.34	0.27	0.20	0.27	13.80	20.10	3.35	2.72	2.04	2.60
Female	80–90	85	0.26	0.16	0.09	0.08	0.06	0.08	7.43	4.46	2.54	2.37	1.67	2.33
Male	40–50	55	0.71	3.47	0.26	0.24	0.20	0.24	8.26	45.83	3.27	3.07	2.59	3.01
Male	40–50	65	0.22	0.62	0.10	0.09	0.09	0.10	5.24	15.58	2.57	2.56	2.47	2.73
Male	40–50	75	0.04	0.09	0.02	0.02	0.02	0.02	3.05	7.71	2.05	2.04	1.89	2.12
Male	40–50	85	0.00	0.02	0.00	0.00	0.00	0.00	1.73	9.38	1.58	1.60	1.53	1.58
Male	50–60	55	1.49	4.23	0.46	0.43	0.32	0.39	12.30	34.85	4.03	3.80	2.71	3.38
Male	50–60	65	0.50	0.99	0.17	0.17	0.13	0.16	8.05	16.32	2.97	2.90	2.25	2.74
Male	50–60	75	0.10	0.18	0.05	0.05	0.04	0.05	4.65	9.42	2.56	2.51	2.13	2.51
Male	50–60	85	0.01	0.03	0.01	0.01	0.00	0.01	2.53	7.78	1.90	1.91	1.63	1.82
Male	60–70	55	2.45	2.76	0.55	0.58	0.47	0.48	15.32	17.23	3.49	3.71	3.00	3.06
Male	60–70	65	0.82	0.68	0.21	0.22	0.16	0.18	9.58	8.36	2.52	2.60	1.89	2.17
Male	60–70	75	0.17	0.20	0.06	0.06	0.05	0.06	5.43	7.20	2.07	2.12	1.63	1.92
Male	60–70	85	0.02	0.03	0.01	0.01	0.01	0.01	2.74	6.19	1.71	1.74	1.60	1.66
Male	70–80	55	5.34	3.02	1.30	1.06	0.66	0.77	24.10	13.85	6.00	4.95	3.17	3.62
Male	70–80	65	2.19	1.30	0.51	0.46	0.36	0.40	16.35	9.65	3.83	3.59	2.79	3.06
Male	70–80	75	0.66	0.53	0.16	0.13	0.11	0.13	10.44	8.39	2.66	2.23	1.81	2.09
Male	70–80	85	0.09	0.06	0.03	0.03	0.02	0.03	5.22	4.47	1.88	1.80	1.21	1.72
Total	40–50	55	11.01	9.56	1.91	1.75	0.65	1.00	41.98	36.36	7.34	6.73	2.48	3.82
Total	40–50	65	4.30	1.54	0.51	0.37	0.27	0.51	25.39	9.18	2.96	2.18	1.58	2.95
Total	40–50	75	1.06	1.97	0.33	0.25	0.19	0.22	12.10	21.67	3.82	2.90	2.24	2.51
Total	40–50	85	0.19	0.09	0.06	0.05	0.03	0.05	7.12	3.48	2.13	1.86	1.21	1.77
Total	50–60	55	0.72	4.80	0.33	0.31	0.32	0.34	8.09	57.95	3.83	3.76	3.89	4.02
Total	50–60	65	0.22	0.65	0.13	0.13	0.18	0.18	4.93	15.28	3.13	3.15	4.10	4.07
Total	50–60	75	0.05	0.09	0.03	0.03	0.03	0.03	3.05	6.24	2.24	2.27	2.14	2.28
Total	50–60	85	0.01	0.02	0.01	0.01	0.01	0.01	1.88	8.65	1.77	1.80	1.89	1.83
Total	60–70	55	1.47	5.74	0.48	0.45	0.30	0.39	11.81	47.86	4.00	3.78	2.53	3.26
Total	60–70	65	0.50	1.16	0.18	0.17	0.13	0.16	7.58	17.95	2.80	2.74	2.01	2.56
Total	60–70	75	0.10	0.16	0.05	0.05	0.05	0.06	4.26	7.56	2.40	2.40	2.22	2.43
Total	60–70	85	0.01	0.03	0.01	0.01	0.01	0.01	2.31	6.72	1.58	1.60	1.38	1.58
Total	70–80	55	3.12	3.93	0.66	0.62	0.48	0.54	18.72	25.25	4.01	3.79	2.96	3.29
Total	70–80	65	1.08	0.94	0.25	0.25	0.18	0.22	11.69	10.59	2.78	2.79	2.02	2.48
Total	70–80	75	0.23	0.23	0.08	0.08	0.06	0.07	6.32	6.88	2.35	2.36	1.81	2.16
Total	70–80	85	0.02	0.04	0.01	0.01	0.01	0.01	2.87	6.50	1.76	1.79	1.77	1.71
Total	80–90	55	4.54	2.52	1.21	1.06	0.77	0.82	20.94	11.87	5.47	4.87	3.72	3.86
Total	80–90	65	1.95	1.25	0.45	0.44	0.34	0.37	14.76	9.39	3.40	3.31	2.56	2.78
Total	80–90	75	0.59	0.53	0.15	0.13	0.10	0.12	9.36	8.21	2.40	2.15	1.63	1.97
Total	80–90	85	0.08	0.07	0.03	0.03	0.02	0.03	4.76	4.88	1.66	1.65	1.18	1.55

*e*_*0*_ life expectancy at birth, *a* starting age of the open age interval, *Classical* classical life table method, *Extrapol.* extrapolation of the death rates using the Gompertz model, *H.-C.* Horiuchi-Coale formula, *H.-C. (hmd)* Horiuchi-Coale formula with the Beta parameter re-estimated on the HMD data, *Mitra* Mitra formula, *Mitra (regr.)* Mitra formula with the mean population age in the open age interval substituted from the indirect estimate (6), *RMSE* root-mean squared estimation error, *Percentage RMSE* root-mean squared estimation errors as percent of values in corresponding full life tables from HMD (i.e., with *a* = 110)

The percentage difference (5) tends to 100% when the first of the methods compared shows much greater errors as compared to the second; it tends to − 100% in the opposite case when the second method performs much worse; it equals zero when the two methods show similar absolute estimation errors.

The estimation errors in the classical method are predominantly positive because of the worldwide growth of the elderly population in the course of demographic transition, which produced population age structures younger (of lower mortality) than the structure of stationary populations assumed in the method. The biases were relatively small (yet, quite substantial) for periods with shorter life expectancy at birth and soared to high levels as life expectancy grew. This comes well in agreement with the formal derivations by Horiuchi-Coale and Mitra. Currently, closing the life table at age 65, both sexes combined, would produce an upward bias in life expectancy at birth as high as 10 years in some countries and more than 2.5 years in many other cases. These errors, with the secular trend of life expectancy increasing by about 2 years per decade (Oeppen and Vaupel 2002; White 2002), correspond to gains of life expectancy over 12–50 years. If data permits closing the life table at age 75 or 85, the estimation errors are down to under 4 or 0.75 years, respectively. Closing the life tables at age 95 yields only minor errors in *e*_0_, while closing at age 55 drives the errors to unacceptably high levels. The errors are generally higher when considering only the female populations or both sexes together.

In Table 1, we also show the estimation errors in life expectancy *e*_*a*_ at the beginning of the open age interval as a percent of the life expectancy from the corresponding full life table. Our interest in this kind of estimation errors is driven by the usual practice in population projections, where the population of the open age interval is projected on the basis of the life table death rate for that age group (Preston et al. 2001). The life table death rate in the open age interval is related to the life expectancy through exactly the same equation (1) as in the classical method. Therefore, the relative error in remaining life expectancy *e*_*a*_ obtained from the classical method shows the relative difference between the actual population death rate for the open age interval and the corresponding life table rate. Hence, the relative errors shown in Table 1 indicate that substituting the actual population change in the open age interval by its estimate, based on the life table death rate, may lead to annual downward biases of dozens of percent in the numbers of deaths in the open age interval.

The extrapolation method, surprisingly, does not improve over the classical method in terms of bias and is rather unstable at open age intervals starting at younger age. Only in cases with high life expectancy and later onset of the open age interval does it systematically outperform the classical method (Fig. 2, plot k). Note that we used the Gompertz model that assumes exponential growth of the death rates as a function of age. If we used a more optimistic logistic-type model (e.g., the Kannisto model (Thatcher et al. 1998) fits better the pattern of mortality deceleration at oldest old age and is used by the WHO and the HMD), the upward biases in life expectancy estimates would be even higher.

The Horiuchi-Coale formula provides a remarkable improvement in terms of estimation errors over both the classical and the extrapolation methods at all levels of life expectancy (Table 1, Figs. 1 and 2, plots f, h, l, m), although the parameters for the formula were estimated back in the 1980s. The vast reduction of estimation errors, after applying the adjustment, indicates that the method is rather robust to violations of its underlying assumptions (the Gompertzian death rates and stable population age structure). Table 1 also includes results for the Horiuchi-Coale formula where we kept the original values for the parameter *α*_*a*_ but re-estimated the other parameter *β*_*a*_ based on our database (“H.-C. (hmd)” columns of Table 1; see Ediev 2017 for the parameter’s values). Updating the model parameters to the more complete HMD provides only a marginal improvement in terms of root-mean squared error (RMSE). Yet, the method’s RMSEs may perhaps be further reduced by fitting the model to more homogeneous data (to groups of populations with similar mortality dynamics and growth histories).

The Mitra formula is generally more accurate than the Horiuchi-Coale method (Table 1, Figs. 1 and 2, plot p), except for female life tables with low values of the life expectancy at birth. However, the method appears to be prone to producing outliers, especially overestimates of life expectancy (Figs. 1 and 2, plots d, g, i, j). The Mitra formula involves the population mean age in the open age interval, $\overline{x}$, an indicator easy to calculate for populations with good-quality data, such as the HMD populations, but problematic for populations with age exaggeration. Hence, we checked if the formula remains accurate after substituting $\overline{x}$ by its prediction based on the regression involving the growth rate and the observed death rate:

6 $$ \overline{x}=C+{k}_1{M_{a+}}^{-1}+{k}_2r{M_{a+}}^{-1} $$

(Ediev 2017). Results for the Mitra formula with the approximate mean age (6) are shown in Table 1, in the “Mitra (regr.)” columns. Substituting the true mean age by its indirect estimate only marginally increases RMSEs. Even based on indirect mean age estimates, the method remains more accurate (but also more prone to producing outliers) than the Horiuchi-Coale method. The differences between the two methods, however, are minor as compared to the errors in the classical method. A closer inspection of cases where the Mitra method produces outlier estimates shows that it is the sensitivity of the method to the population growth rate that makes it unstable. Apparently, the quadratic (with respect to *r*) term in (3) causes the method to produce strong overestimates of the life expectancy in cases with strong population growth or decline and when the population stability assumption is violated.

In all methods, errors tend to increase as life expectancy grows. Interestingly, all methods tend to err more often to the positive side, i.e., they overestimate life expectancy, although the non-classical methods are free from the classical method’s sources of error that is nested in the stationarity assumption. The reasons for the positive errors are different among the methods. The extrapolation method produces positive or negative errors depending on whether the death rates increase steeply above or below the minimal age of the open age interval. Mortality acceleration at younger old ages (Horiuchi and Wilmoth 1997;Horiuchi 1997), which is more typical in female populations, explains positive biases in the extrapolation method. At the same time, mortality deceleration at older ages (Horiuchi and Wilmoth 1997, 1998; Horiuchi et al. 2003) may explain somewhat more prevalent negative biases of the extrapolation method at the open age interval 85+ and the tendency of the method to produce negative errors for the open age interval 95+ (results not shown here). The Horiuchi-Coale and Mitra methods tend to produce positive errors, because protracted periods of mortality decline at old age, as observed in many countries, and produce population age structures even younger than the stable populations assumed in the two methods (Ediev 2014; Guillot 2003; Horiuchi and Preston 1988). The Mitra method, additionally, tends to overestimate the true life expectancy because of the aforementioned instability of the method in cases of strong population change.

The tendency of both the classical and the Mitra estimates to exaggerate the life expectancy suggests a novel combined approach when the life expectancy estimate is obtained as the minimum of the two estimates:

7 $$ {e}_a=\operatorname{MIN}\left({e_a}^{\mathrm{Clas}.},{e_a}^{M.}\right) $$

**Fig. 3:**
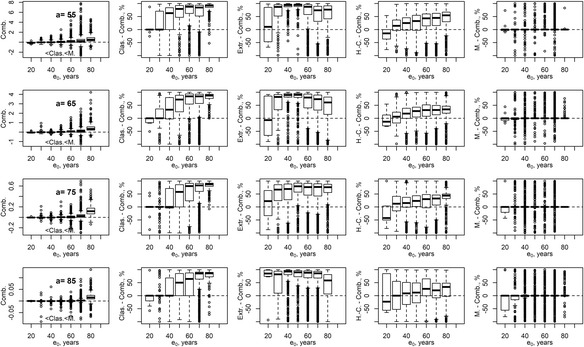
Boxplots of estimation errors in life expectancy at birth obtained by the combination (7) of the classical and Mitra methods (the combined method, “Comb.”) at selected starting ages *a* of the open age interval (plots in the first column). Plots in the last four columns: boxplots, as a function of life expectancy at birth, of percentage differences of absolute errors (5) of the combined method and single methods as indicated at the vertical axes. Female populations. Methods covered: the classical method (“Clas.”), extrapolation method based on 20-years-long age base (“Extr.”), Horiuchi-Coale method (“H.-C.”), and Mitra method (“M”)

Here, upperscripts “Clas.” and “*M.*” refer to the classical and Mitra estimates. Estimation errors of life expectancy at birth in the combined method (7), also in comparison to single estimation methods, are presented in Fig. 3. Taking the minimum of the two estimates helps avoid the outlier estimates of the Mitra method (compare the boxplots in the first column in Fig. 3 at *a* = 75 and *a* = 85 to the boxplots “d” in Figs. 1 and 2 for the Mitra method). At the same time, the combined method performs, in most of the cases, as good as the Mitra method (column 5 in Fig. 3) and outperforms all other single-method alternatives (columns 2–4 in Fig. 3).

Our above results show that both the Horiuchi-Coale and Mitra formulas perform well in reducing life expectancy estimation errors caused by aggregating data for the open age interval. The Mitra method is marginally more accurate but less stable. Its reliance on the possibly exaggerated mean population age may be overcome by using the indirect estimates of the mean age (6). Both methods are by far superior to the classical and the extrapolation methods. It is rather surprising that the authors of these two methods came up with contradicting results in their papers.

A closer examination of the original papers, however, shows that a large part of the numerical differences between Horiuchi-Coale and Mitra were, in fact, due to different inputs used in their calculations rather than methodological differences. In particular, the largest discrepancy in the original papers was for *e*_65_ for El Salvador in 1961: Mitra’s estimates were larger 3.12 years for women and 3.02 years for men. When we recalculated the life expectancies using similar inputs in both approaches (Mitra 1984, pp. 11–12), we found that the two approaches are more consistent: Mitra’s formula gives estimates by 1.69 and 1.27 years larger, respectively. After our recalculation, the estimates become closer also for Canada, Japan, Switzerland, UK, Mexico, and Malaysia. Altogether, the two methods differ by more than 1 year in only two cases, El Salvador and Puerto Rico, out of 13. If one takes into account that the Mitra method was relying on potentially biased official estimates of the mean population age in the open age interval, it becomes clear that the authors were more consistent than they concluded.

## Discussion

Our results show that the violation of the stationary population assumption of the classical life table method has strong consequences for the accuracy of life expectancy estimates. The errors in the classical method increase as mortality declines. For a currently low-mortality population, closing the life table at age 65 would produce an HMD-average upward bias of more than 3 years and even larger RMSEs in the life expectancy at birth calculated by the classical method. Continuing increases in life expectancy will drive the biases of the classical method to even higher levels.

The methods developed by Horiuchi-Coale and Mitra drastically reduce estimation errors in the expectation of life as compared to both the classical and the extrapolation methods. Wider usage of these methods should be encouraged for populations where data on old-age mortality are missing (for example, due to small population size) or corrupted by age exaggeration or age misreporting in general. Although estimation errors of all methods increased as life expectancy grew for HMD populations, the comparative advantage of the Horiuchi-Coale and Mitra methods has only strengthened over time. Among the methods considered, the Horiuchi-Coale method may be preferred as being close to the best (on average) estimates provided by the Mitra formula but being more stable. Even better results, however, may be obtained by considering several alternative methods and selecting the most reasonable estimate (see the combined method (7) for an example).

A wider usage of the Horiuchi-Coale and Mitra methods in demographic analysis may be facilitated by their perfect fit to a number of popular indirect demographic methods. The Brass Growth Balance method, the Preston and Coale method, the Hill Generalized Growth Balance method, and the Bennett and Horiuchi Synthetic Extinct Generations method all involve estimates of the population growth rate (Moultrie et al. 2013; United Nations 1983). These methods provide ready inputs for the Horiuchi-Coale and Mitra formulas.

The method of extrapolating the death rates into the open age interval does not appear to be a good alternative to the classical method. The WHO and the HMD use an S-shaped model for extrapolating the death rates as alternative to the J-shaped Gompertz model used here. Indeed, logistic-type models were shown to better fit the deceleration of mortality at oldest old ages (Missov et al. 2016; Thatcher et al. 1998). However, our results for the younger open age intervals, where all mortality models fit closely to each other, suggest that the conclusion about the inferiority of extrapolation to the Horiuchi-Coale and Mitra methods is, in general, applicable to any mortality models other than the Gompertz model. In fact, a logistic-type model might even accentuate the upward biases in life expectancy estimates. In many applications, however, it is important to extend the age profile of the death rates into the open age interval. Although our results discourage from using the popular extrapolations, one may combine the more accurate adjusted estimate of life expectancy *e*_*a*_ with the extrapolation model by constraining the parameters of the latter to fit the life expectancy estimate (Ediev 2017). Another area for further work is the study of estimation errors in non-parametric methods for the open age interval not considered here (Camarda 2012; Currie et al. 2004; de Beer 2012; Kostaki and Panousis,^2001^; Rizzi et al. 2015).

As an important consequence of the discrepancy between the actual death rate and life expectancy for the open age interval, the traditional approach of projecting the population in the open age interval (Preston et al. 2001) may lead to overestimates by dozens of percent of deaths in the open age interval. The adjustment formulas considered here as well as related conditioned extrapolations of the death rates may be used to compensate for this projection bias.

One may further improve life expectancy estimates by fitting the models on populations with closer history of growth and mortality reduction (e.g., of regions of a given country). Another direction of improvement might be considering population models more advanced than the stable population. For example, one may consider effects of the changing growth rate and mortality on the population age composition (Brouard 1986; Ediev 2014; Guillot 2003; Horiuchi and Preston, 1988). Results for the method combining the classical estimates with the Mitra method also suggest that pooling together several methods and making use of expert judgment about the likely direction of estimation biases may help reducing the estimation errors and stabilizing the estimation results in particular country cases.

## Footnotes

1 HMD modifies (smooths and extrapolates) the original data at some ages above age 80 using the logistic model. This might have distorted our findings for the highest open age intervals, especially for the extrapolation method. Yet, after re-running our calculations on the raw, not modified, death rates also provided in the HMD, we came to results similar to those presented in this paper. Root-mean squared error (RMSE) obtained on raw data differ only in the second digit after comma from the RMSEs presented in Table 1 for all methods, except for the extrapolation. Even for the extrapolation method, there was only one case when RMSE based on the raw data differed in the first digit after comma from the results presented in Table 1 and was substantial in relative terms: females, life expectancy at birth range 40–50 years, *a* = 85 (RMSE on the smoothed death rates, 0.04; RMSE on the raw data, 0.16). Data were downloaded on 12.02.2016 and are consistent with inputs in (Ediev 2017).
